# Understanding the Impact of Chronic Obstructive Pulmonary Disease and Intervening to Improve Self-Management in the Context of Multi-morbidity

**DOI:** 10.5334/ijic.4187

**Published:** 2018-07-19

**Authors:** Sameera Ansari

**Affiliations:** 1 UNSWORKS, UNSW Sydney, New South Wales, AU

**Keywords:** COPD, multimorbidity, self-management, primary care, patient activation, implementation science

## Background

Though the whole (multi-morbidity) is more than the sum of the parts (co-morbidities), it is sometimes necessary to consider a specific index condition during patient-provider consultations. The index disease prioritised by patients depends on which chronic health condition is predominant in their lives at a given point of time. Chronic obstructive pulmonary disease (COPD) was the index condition studied in the context, or backdrop, of multi-morbidity in this thesis.

## Objectives

This thesis comprises two phases of original research on self-management of COPD in the context of patients’ multi-morbidity. Both phases of research were conducted in Sydney, Australia.

Phase I was a qualitative study which aimed to:

- Explore the impact of a new diagnosis of COPD among primary care patients in the context of their multi-morbidity.
- Gain an understanding of how the patients incorporated COPD into their lives.

Phase II comprised development, implementation and evaluation of a tailored, self-management education program for patients with COPD in the context of multi-morbidity in general practice. The study, titled the **A**ctivating **P**rimary Care **CO**PD Patients with **M**ulti-morbidity (APCOM) pilot project, hypothesised that at six months’ follow-up after the program, participating patients would have:

- Better activation in terms of their COPD-related health behaviour.
- Improved knowledge and self-management capacity of COPD.
- Increased self-efficacy in terms of their overall health behaviour.

## Methods

Phase I comprised of semi-structured interviews with 17 patients who were recently diagnosed with COPD and had existing co-morbidities. The interviews were inductively coded and thematically analysed using a constructivist approach.

Based on the findings of Phase I as well as a gap in extant literature, the self-management education program was developed and piloted in Phase II. The program was designed around the Health Belief Model, a widely-tested model of human behavioural change. Participating practice nurses (PNs) were trained in one-day workshops and provided with research support throughout the program. The program was tailored and delivered to participating patients (N = 50) by their PNs in individual face-to-face sessions using motivational interviewing. The PNs followed up with the patients for five months after the last session via a monthly phone call.

The impact of the program was evaluated by the Patient Activation Measure (PAM 13), COPD Knowledge Questionnaire (COPD-Q), COPD Assessment Test (CAT), Multimorbidity Illness Perceptions Scale (MULTIPleS), a COPD-specific version of MULTIPleS, Morisky Medication Adherence Scale (MMAS-8) and accuracy of inhaler device technique. A qualitative process evaluation of the program was conducted via semi-structured interviews with 10 PNs, 7 general practitioners (GPs) and 12 patients. The interviews were thematically analysed using a constructivist approach and interpreted through tenets of the Normalisation Process Theory.

## Findings

Phase I: Most participants had difficulty recognising the salience of COPD and its long-term implications despite accepting the diagnosis. Self-management capacity and relevant healthcare utilisation were challenged by low prioritisation of COPD due to their multi-morbidity.

Phase II: Paired comparison of the endpoints of 44 patients who completed six months’ follow-up showed a significant improvement in patient activation (p < 0.001), COPD knowledge (p < 0.001), clinical impact of COPD (p = 0.012) and accuracy of inhaler device technique (p = 0.001). There was no significant change in the patients’ perception of their multi-morbidity (0.822) and COPD-related multi-morbidity (0.084), or their medication adherence (0.139), following the program.

The qualitative process evaluation yielded mostly positive feedback on acceptability, feasibility and sustainability of the program. All PNs found that participating in the study improved their confidence and skills for COPD management. They were satisfied about better health-related outcomes for many patients following the program. The main challenges faced were scheduling of education sessions, contacting the patients for monthly follow-ups and complications presented by the patients’ co-morbidities. The GPs recognised the significance of the program and perceived it to be beneficial for participating patients. All but one GP were supportive of upskilling PNs for providing health education.

Overall, the patients perceived the program as a positive and worthwhile experience. They were more aware of COPD and its implications following the program. Most patients had better adherence to their prescribed medication following the program. Many patients made changes to their lifestyle, including enhanced physical activity and better dietary intake. The program enabled some patients to cope with health-related stressors in the face of their multiple chronic conditions.

## Conclusions

The findings from Phase I established a need for personalised interventions to enhance self-management of primary care patients with COPD in the face of multi-morbidity. The APCOM pilot study in Phase II found that the innovative self-management education program was successful in improving health-related self-efficacy of the participating patients. These findings form an evidence base for upscaling the program to be tested in a randomised controlled trial.

## Implications for integrated care

Phase I demonstrated an insight into how patients with existing chronic conditions cope with diagnosis of another condition and incorporate its management into their lives. Phase II developed and implemented a complex clinical intervention for self-management of chronic disease in day-to-day general practice. The study promoted patient-centred holistic care and promoted shared decision-making with their PNs and GPs.

The results presented in this article are based on the author’s thesis completed at UNSW Sydney, NSW, Australia in January 2018.

